# Covert Intention to Answer to Self-Referential Questions Is Represented in Alpha-Band Local and Interregional Neural Synchronies

**DOI:** 10.1155/2019/7084186

**Published:** 2019-01-06

**Authors:** Jeong Woo Choi, Kwang Su Cha, Kyung Hwan Kim

**Affiliations:** ^1^Department of Biomedical Engineering, Yonsei University, Wonju 26493, Republic of Korea; ^2^Department of Neurosurgery, University of California, Los Angeles, CA 90095, USA

## Abstract

The most fundamental and simplest intention for interpersonal communication may be the intentions to answer “yes” or “no” to a question, based on a binary decision. However, the neural mechanism of this type of intention has not been investigated in detail. The main purpose of this study was to investigate cortical processing of the “yes/no” intentions to answer self-referential questions. Multichannel electroencephalograms (EEGs) were recorded while covertly answering self-referential questions with either “yes” or “no”. Event-related spectral perturbation (ERSP) and interregional phase synchrony (PS) were investigated to identify the differences in local and global neural synchronies between two intentions. We found that the local and interregional neural synchronies in the alpha-band were significantly different between “yes” and “no,” especially at the period of retaining the intention in mind, which was greater for “no” than for “yes.” These results can be interpreted to signify that a higher cognitive load during working memory retention or higher attentional demand is required for the “no” intention compared to “yes.” Our findings suggest that both local and global neural synchronies in the alpha-band may be significantly differentiated during a critical temporal epoch, according to the contents of the mental representation of the intention.

## 1. Introduction

Intention is the basis of actions and is critical in decision making and communication with others. Recent neuroimaging studies have shown that a variety of regions in the human brain are associated with intention [1–3]. Lau et al. [3] showed the activation of the presupplementary motor area during representation of motor intention for a self-paced finger movement. Haynes et al. [2] showed that the medial and lateral prefrontal cortices encode a subject's covert intention to choose one of two possible mental arithmetic tasks. A magnetoencephalogram study showed that Broca's area and parietal region control the intention to speak [1]. These divergent findings suggest that intention entails a variety of themes, and thus, only a specific type of intention should be investigated by a neuroimaging study. A simple and fundamental model of intention should be set up beforehand.

The most fundamental and simplest intention may be on responding to a question by either “yes” or “no,” based on a binary decision. By answering “yes” or “no,” we can communicate with others, i.e., deliver our feelings, thoughts, and private/general information. The “yes/no” answer can be an important communication tool for the patients with completely locked-in syndrome (CLIS) [4], and decoding the “yes/no” intentions was suggested as a means to develop a brain-computer interface (BCI) [5]. However, the neural mechanisms of this simple and fundamental intention have not been investigated in detail.

The electroencephalogram (EEG), especially event-related potential (ERP), is suited to investigate cortical dynamics during a cognitive task due to its high temporal resolution. In a recent ERP study, we identified that the integration of semantic and autobiographical information processing precedes answering “yes” or “no” in response to self-referential questions [6]. The posterior N400 (at ∼300–500 ms) was interpreted to reflect this integrated information processing. We also expected that the decision and the intention to answer are generated simultaneously with or immediately after this integrated information processing. Based on this, we hypothesized that the brain activities reflecting the intention to answer “yes” or “no” in working memory (WM) can be identified during a temporal period, after the integration of semantic and autobiographical processing, and before the actual covert answer. Here we call this “a period of retaining intention in mind”. To verify our hypothesis, here we explore the difference in event-related brain activities between answering “yes” and “no” in response to self-referential questions, especially at the period of retaining intention in mind. In addition, we tried to compare our findings with the control condition, which was required only to read but not to answer the presented question so that the intention to answer would not be generated.

The investigation of neural synchrony may provide valuable insights into cortical information processing beyond conventional ERP analysis in the time domain. In particular, the local neural synchronies in the alpha-band (8–13 Hz) are known to reflect cortical inhibition [7] and idling [8]. Previous studies showed that the reductions of local alpha-band power were reduced in response to stimulus presentation of various sensory modalities [7]. Beyond sensory perception, it has been also reported that increases in alpha-band neural activity were induced by cognitive function such as working memory (WM). For example, when subjects retained visually presented items in mind, the alpha-band power was increased as a function of the number of items, which means that the alpha event-related synchronization (ERS) is associated with WM load [7, 9–11]. Klimesch et al. [7] suggested that this alpha ERS reflects an active top-down inhibitory control to prevent the interference due to the retrieval of previously encoded memory.

Interregional phase synchronization (PS) signifies the functional integration of the widely distributed neural assemblies in task-relevant cortical regions, whereas the ERSP is due to the synchronization of localized neural activities [12, 13]. Interregional PS in the alpha-band was recognized to be involved in the formation of a unified mental construct, such as a mental image, a hypothesis, a planned action, or a thought [14]. It is also known that the interregional alpha-band PS is associated with the WM load [11, 15], as well as the local alpha ERSP. Palva et al. [15] showed that the alpha-band PS between frontal and parietal regions were increased as WM load increased and interpreted that the alpha-band PS is involved in the top-down modulation for object representation in WM.

We consider that the judgement of “yes” or “no” in response to a question results in a unified mental construct. Also, the cognitive load during retaining the intention to answer in mind might be different between “yes” and “no” based on several psychophysical studies which showed that saying “no” requires more effortful reconsideration after comprehending a sentence [16, 17]. Thus, we expected that noticeable differences between “yes” and “no” would be identified in both local and global neural synchronies in the alpha-band, during the period of retaining intention in mind.

The purpose of this study was to find the neural signatures of “yes” and “no” intentions in response to self-referential questions, for the first time to our knowledge. To this end, we investigated both local and global neural synchronies in the alpha-band, focusing on the differences between the “yes” and “no.” We expect that this study can provide a basis to study how different contents of mental representation of intention are differentiated in cortical activities.

## 2. Materials and Methods

### 2.1. Subjects

25 subjects without record of neurological or psychiatric illness were enrolled in the experiment (age: 23.12 ± 2.93 years, 13 males). All the subjects were undergraduate students of Yonsei University, and native Korean speakers, and right-handed. The experimental protocol was approved by Yonsei University Wonju Institutional Review Board (IRB). All experiments were performed in accordance with the Declaration of Helsinki and the guidelines and regulations of the IRB. Written informed consent was obtained from each subject before the experiment.

### 2.2. Experimental Task

Subjects completed a written report on their autobiographical facts such as first name, last name, age, gender, and job, prior to the experiment. Forty pairs of questions and answers were generated for each subject based on the report. All the questions were composed of 2-3 Korean words. One question in each pair was in accordance with the subject's identity and thus should be answered “yes.” The other did not agree with the subject's identity and thus should be answered “no.” The two questions in each pair were identical except for one critical word (underlined and italicized letters in the example below). Below, two questions are presented as examples of questions (translated to English from Korean) for the case where the subject's true job is student:

  Type (a), without autobiographical fact violation: Is your job a *student*?
  Type (b), with autobiographical fact violation: Is your job a *professor*?

The number of words in each question was either two or three, and the average number of characters in each critical word was 3.18 ± 1.02.

The experimental procedure included two response conditions: (1) covert response condition, where the subjects had to silently respond to the questions without verbalizing the responses and behavioral response, and (2) nonresponse condition, where they just watch and pay attention to the presented words without any response. All the words were presented as white letters on a black background screen. The size of each character was 3.3 cm (width) by 4.27 cm (height). Commercially available software was used to present the word stimuli (PRESENTATION; Neurobehavioral systems, Berkeley, CA, USA). All the subjects were comfortably sitting on a chair in front of a computer monitor and instructed to pay attention to the visually presented words. The distance between the subjects' eyes and the monitor was approximately 75 cm. Each word of a sentence appeared sequentially on a 17-inch computer monitor one by one.

**Figure 1:**
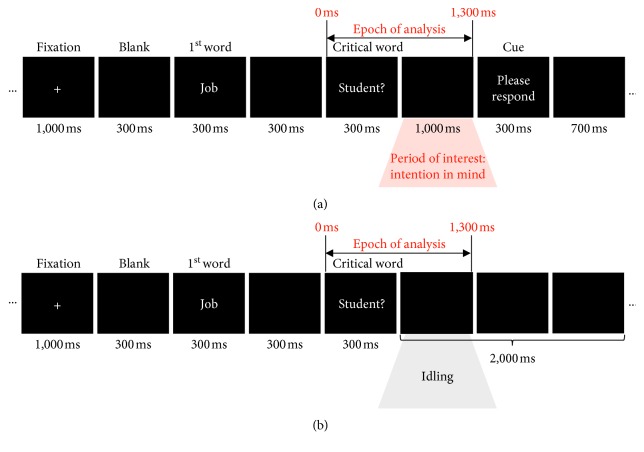
Experimental paradigms for the (a) covert response condition and (b) nonresponse condition.

The procedure of the covert response condition is illustrated in Figure 1(a). A fixation mark (“+”) was presented for 1000 ms, which was followed by a black screen for 300 ms, and then, each word in the question was presented in turn for 300 ms. A black, blank screen was presented for 300 ms between the words. A blank screen followed the last, critical word for 1000 ms. Finally, as the cue stimulus for the response, “Please respond” (in Korean) was presented for 300 ms to notify the subjects to respond covertly with either “yes” or “no” without any behavioral response. A black blank screen was presented for 700 ms between the cue stimuli and the onset of the next trial. The average duration of each single trial was 4380 ± 274.95 ms. Before starting the covert response condition, all the subjects were instructed not to answer immediately to the questions, but to retain the decision of the answer, “yes” or “no,” in their mind until the “Respond” cue appears. This would allow us to investigate the cortical activity during retaining the intention to answer “yes or “no” in WM (or short-term memory).

We conducted the nonresponse condition as a control condition, in which the information on the answer, yes or no, is not represented in mind, even though the response might be generated automatically. This was to investigate the changes in brain activities according to whether the information retention exists or not. In the nonresponse condition, the procedure of presenting questions was same as that for the covert response condition as shown in Figure 1(b). The difference lies after the last critical word, which was followed by only a blank black screen for 2000 ms. Here, the subjects were requested to simply read the presented questions in their mind without any mental or behavioral responses. Thus, it is expected that the intention to answer either “yes” or “no” was not represented in mind, i.e., in the WM in the nonresponse condition.

Both response conditions may induce automatic answer immediately after the last word since they all end with a question mark as shown in Figure 1. However, the most crucial difference between the two response conditions is whether the intention on the answer would be retained in mind for a short period or not. Thus, the temporal period of interest for further analyses was ∼0-1 sec interval after the critical word, which is expected to be a period of retaining the intention in mind before the cue “Respond” appears only in the covert response condition (as denoted by a red shade in Figure 1(a)). For the nonresponse condition, this period is just for the idling, as denoted by a gray shade in Figure 1(b).

The overall experiment was divided into two blocks for each condition. Fifty questions for “yes” answers and 50 questions for “no” answers were randomly presented in each block. Each block took 7.3 minutes in average, and a 5 min resting period was given between blocks. Nonresponse conditions were conducted before the covert response conditions, to prevent involuntary and automatic responses which might be induced if the nonresponse condition were presented after performing the covert response condition.

### 2.3. Electroencephalogram (EEG) Recording and Data Analysis

The 60-channel EEGs were recorded at a sampling rate of 500 samples/s, based on the 10-10 system, along with a vertical electrooculogram (EOG) at a site below the right eye, using an EEG recording system (Brain Products GmbH, Munich, Germany). An electrode cap with 60 sintered Ag/AgCl electrodes was used (EASYCAP, FMS, Munich, Germany). The impedances of all the electrodes were reduced below 10 kΩ. The reference electrode was formed by linked mastoid electrodes, and the ground electrode was placed between Fpz and Fz. A bandpass filter (0.03–100 Hz) and a notch filter (60 Hz) were applied to reduce background noise.

Single-trial waveforms were obtained during the −500∼1300 ms interval after the critical word onset. Severely contaminated single-trial waveforms were eliminated from further analysis if they included nonstereotyped artifacts such as drifts. Stereotyped artifacts such as ocular and muscular artifacts were corrected using an independent component analysis (ICA) described by Jung et al. [18]. In addition, if the absolute value of the EOG exceeded ±100 *μ*V, the corresponding segments of the EEG were removed from further analysis. For the covert response condition, the number of epochs per subject was 98.92 ± 2.97 and 98.04 ± 5.64 for “yes” and “no,” respectively, and those for the nonresponse condition were 95.88 ± 10.66 and 96.00 ± 10.09 for “yes” and “no,” respectively.

EEGLAB (https://sccn.ucsd.edu/eeglab/index.php), an open source toolbox, was used for the preprocessing such as segmentation of single-trial EEGs, artefactual trial elimination, and ICA-based artifact removal [19]. Custom MATLAB scripts were written and used for all other analyses such as ERSP, interregional PS, and statistical analyses.

### 2.4. Event-Related Spectral Perturbation (ERSP) Analysis

The temporal profile of spectral characteristics was examined by an ERSP analysis using a continuous wavelet transform (CWT) based on a complex Morlet wavelet [20]. The number of cycles for the CWT linearly increased according to the frequency from 4 to 13.5, at the lowest (1 Hz) and the highest frequencies (100 Hz), respectively [19]. This method provides better frequency resolution at high frequencies, and it is better matched to the linear scale that we adopted to visualize the time-frequency map [19]. The induced spectral power was calculated by averaging the ERSP patterns of each single trial [21]. The time-frequency distribution of ERSP patterns was represented as the ratio of the relative change to the power in a baseline interval from −300 to 0 ms prior to stimulus onset, to reduce intersubject variability and to normalize power changes across different frequency bands.

We employed mass-univariate approach with cluster-based permutation test for correcting multiple comparisons [22] in order to find the time, frequency, and electrode showing significant differences between “yes” and “no” without a priori knowledge. The analysis begins with the distribution of power differences between “yes” and “no” in the three-dimensional (3D) time-frequency-electrode space, for two response conditions. From the two distributions of “yes-no” power differences in 3D space, we obtain the distribution of *t*-values in 3D space by paired-sample *t*-tests. This yields the subregions in 3D space corresponding to high “yes-no” difference and significant difference between response conditions. The time, frequency, and electrodes determined from this procedure forms the “region of interest” in which the neural activities are differentiated between “yes” and “no,” and the “yes-no” difference is differentiated between two response conditions. Overall procedure consists of three major steps, (1) finding significant electrodes, (2) finding significant time-frequency region, and (3) performing a post hoc test between “yes” and no” if necessary.

Step (1) enables to reduce the distribution of *t*-values to the one in two-dimensional space (i.e., time-frequency) so that the number of multiple comparisons is greatly reduced [22]. The power difference between “yes” and “no” is calculated at all the points in the 3D space for each response condition for each subject. Then, the distribution is subject to multiple *t*-tests for the comparison between two response conditions. The number of *t*-tests is determined by the number of points in 3D space, which is 407,160 = 261 × 26 × 60 since there were 261 time samples (0–1300 ms), 26 frequency points (5–30 Hz), and 60 electrodes. The *t*-values are averaged within the time-frequency window selected by visual inspection of the time-frequency map, yielding the spatial distribution of *t*-values. And then, the electrodes showing low *p* values below a predetermined threshold (0.05) are selected.

Since we have reduced the problem to 2D space, we try to find significant clusters in the time-frequency space, we try to find significant clusters in the time-frequency space in step (2). The distribution of power difference in time-frequency space is obtained by averaging over the electrodes determined in step (1). Then, similarly to step (1), the power differences are statistically compared between two response conditions at all time-frequency points by paired-sample *t*-test. The number of comparisons is 6,786 = 261 × 26. The time-frequency points with low *p* values below a predetermined threshold (0.01) are first screened, and then, the clusters are formed if more than two successive points are screened along either time or frequency axis. The *t*-values within a single cluster are summed to calculate the mass of the cluster, *t*_mass_. The significance of a cluster is determined by comparing the *t*_mass_ with its null distribution which is obtained by surrogate data. The null distribution of *t*_mass_ was obtained from the largest values of *t*_mass_ for each of 5,000 surrogate data, which were derived by random permutation of response conditions. Finally, a time-frequency cluster is determined to be significant if its *t*_mass_ is above the highest 5% of the null distribution.

In the step (3), the difference between “yes” and “no” is investigated by post hoc pairwise comparisons within the significant subregion in 3D space. The averaged power over the electrodes and the time-frequency ranges are statistically compared between “yes” and “no” for each response condition by paired-sample *t*-test with Bonferroni correction.

### 2.5. Phase Synchronization (PS) Analysis

Weighted phase lag index (WPLI) was computed among all electrode pairs as a measure of interregional PS [23]. The WPLI is recognized to be insensitive to spurious synchronization due to the volume conduction, and robustly insensitive to noise. It has also been shown that the WPLI is better in detecting true synchronization than imaginary coherence (ImC) and phase lag index (PLI) [24, 25]. To reduce the large bias due to small sample size, a debiased WPLI (dWPLI) estimator was used to estimate the squared WPLI as suggested by Vinck et al. [23]:

(1) Ω^W=∑j=1N∑k≠jNImXjImXk∑j=1N∑k≠jNImXjImXk,

where *N* represents the total number of trials and Im{*X*} denotes the imaginary part of cross spectrum between two single-trial EEGs. The dWPLI ranges from zero (no synchronization) to one (maximum synchronization).

The dWPLI was calculated for all possible electrode pairs (*N*=210) of 21 sparsely selected electrodes (Fp1, Fpz, Fp2, F7, F3, Fz, F4, F8, C3, Cz, C4, T7, T8, P7, P3, Pz, P4, P8, O1, Oz, and O2). The cross-spectrum of single-trial EEGs was calculated using fast Fourier transform (FFT) and 500 ms Hanning windows for every 10 ms (i.e., 490 ms overlap) from −250 ms to 1750 ms relative to the critical word onset time (frequency resolution: 1 Hz). The dWPLI values at every time and frequency point for each electrode pair can be illustrated as a time-frequency map. We also obtained the averaged time-frequency map of dWPLI values over all electrode pairs.

To find the region in time-frequency space showing the significant “yes-no” difference, we employed cluster-based permutation test similarly to the ERSP analyses. First, the difference of dWPLI was statistically compared between “yes” and “no” at all the points in time-frequency (TF) space by paired-sample *t*-test for each response condition. The number of comparisons was 3,406 = 131 × 26 (0–1300 ms with 10 ms temporal resolution and 5–30 Hz with 1 Hz frequency resolution). The time-frequency points with high *t*-values above a threshold (*t* = 1.71) are first determined, and then, the clusters of TF points are formed if they are selected successively either along time or frequency axis. From a null distribution of the cluster mass (*t*_mass_) generated by 5,000 surrogate data, a TF cluster is determined to be significant if its *t*_mass_ is above the highest 5% of the null distribution. The mean values of the dWPLI within the determined TF cluster were statistically compared using paired-sample *t*-test at each electrode pair. Then, the *p* values by multiple comparisons were corrected across all electrode pairs so that the false discovery rate (FDR) was less than 0.05 [26].

## 3. Results

### 3.1. Event-Related Spectral Perturbation (ERSP) Analysis

**Figure 2:**
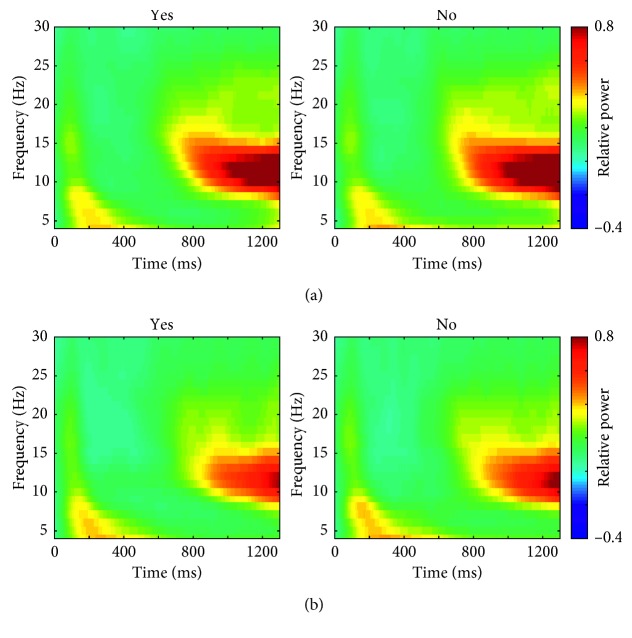
Time-frequency patterns of event-related spectral perturbation (ERSP) in lower frequency bands (<30 Hz), averaged over all electrodes for the (a) covert response condition and (b) nonresponse condition.

Figures 2(a) and 2(b) show the time-frequency patterns of the induced (nonphase locked) neural synchronies for the covert response and nonresponse conditions, respectively. All the time-frequency maps were obtained by averaging over all electrodes. The increase of theta-band activity and the decrease of alpha-band activity with a slight delay were observed in early period (0–400 ms), and the alpha-band activity was increased once again at 800–1300 ms period in 8–13 Hz. The patterns of time-frequency distribution were common for both “yes” and “no” for both response conditions (Figures 2(a) and 2(b)), and they seem to be comparable to a typical pattern of oscillatory cortical activities in response to external stimuli which was suggested in [27].

**Figure 3:**
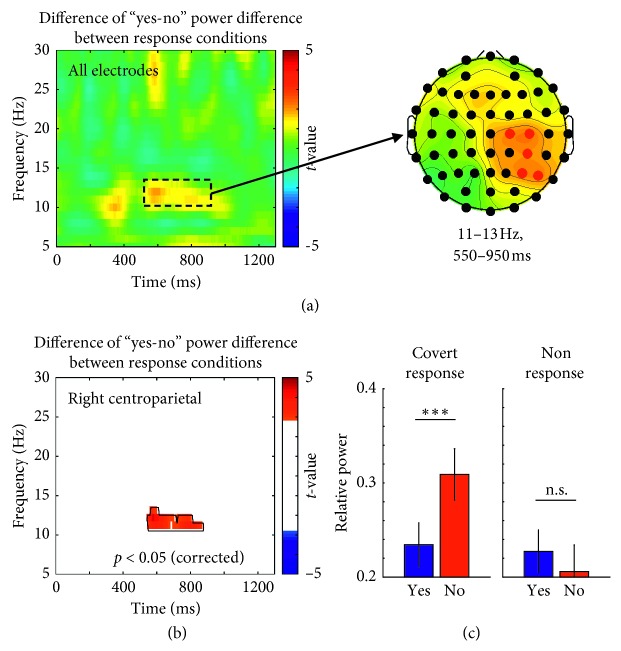
(a) Time-frequency representations of the variation of “yes-no” power difference according to response condition and its topographical distribution (red dot: selected five electrodes). (b) Significant variation of “yes-no” power difference according to response condition (*p* < 0.05, corrected by cluster-based permutation test). Black contour denotes the time-frequency cluster showing significant difference. (c) Post hoc pairwise comparisons of the mean power within the significant cluster between “yes” and “no” for each condition (^*∗∗∗*^: *p* < 0.001, n.s.: nonsignificant, by paired-sample *t*-test with Bonferroni correction).

Figure 3(a) shows the time-frequency pattern and topographical distribution of the variation of “yes-no” power difference according to the response condition. The *t*-values for each time-frequency-electrode bin were obtained by paired-sample *t*-test of “yes-no” power differences between two response conditions. The higher and positive *t*-value means that the “yes-no” power difference is stronger for the covert response condition than the nonresponse condition. The strong variation of “yes-no” power difference according to response condition was found in the upper alpha-band (11–13 Hz) at in 550–950 ms (left panel of Figure 3(a)), especially at the electrodes located over right centroparietal region (right panel of Figure 3(a)). Within the TF subwindow, five electrodes (C2, C4, CP4, P4, and P6) in the right centroparietal region showed the strongest variation of “yes-no” power difference according to response condition (denoted by red dots in the right panel of Figure 3(a)). Thus, those electrodes were selected to remove the independent variable “electrode” and reduce the problem to find the significant time-frequency range (as described in Materials and Methods section).

The variation of “yes-no” power difference according to response condition was significant within a single time-frequency range around 545–880 ms in the upper alpha-band (11–13 Hz) (as denoted by a solid contour in Figure 3(b)). When the mean power within this significant contour was compared between “yes” and “no” within each response condition by post hoc test, the alpha-band power was significantly different only in the covert response condition, which was stronger for “no” compared to “yes” (Figure 3(c)), *t*(24) = −3.76, *p* < 0.001 for the covert response condition, and *t*(24) = 0.86, *p*=1 for the nonresponse condition with Bonferroni correction.

### 3.2. Phase Synchronization (PS) Analysis

**Figure 4:**
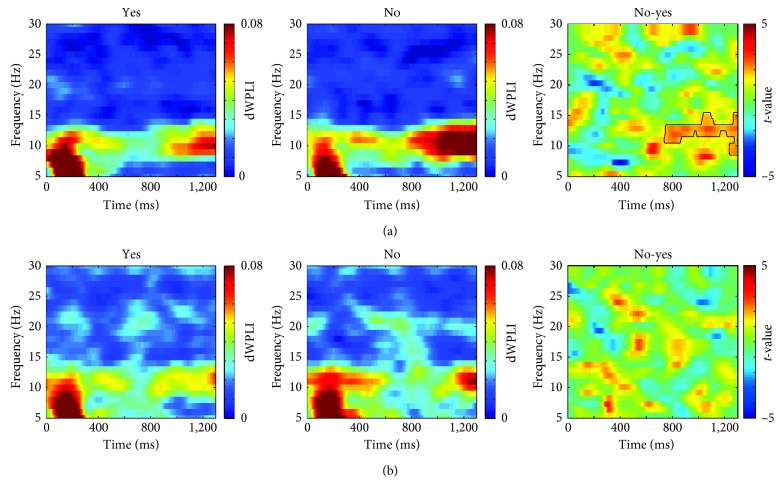
Time-frequency representations of the interregional phase synchrony (PS) in the lower frequency bands (<30 Hz) were obtained by the dWPLI values over all possible electrode pairs (*N*=210) for (a) covert response condition and (b) nonresponse conditions. The black contour in the rightmost panel in (a) denotes the time-frequency cluster showing significant difference (*p* < 0.05, corrected by cluster-based permutation test).

Figure 4 shows the time-frequency representations of interregional PS averaged over all possible electrode pairs (*N*=210). The dWPLI increased in the theta-band in the early period (0–300 ms) but increased in the alpha-band in the late periods (800–1300 ms). The significant difference between “yes” and “no” was identified only in the alpha-band during the late period, especially for the covert response condition, as denoted by the black contour in Figure 4(a). No significant difference between “yes” and “no” was found for the nonresponse condition (Figure 4(b)).

**Figure 5:**
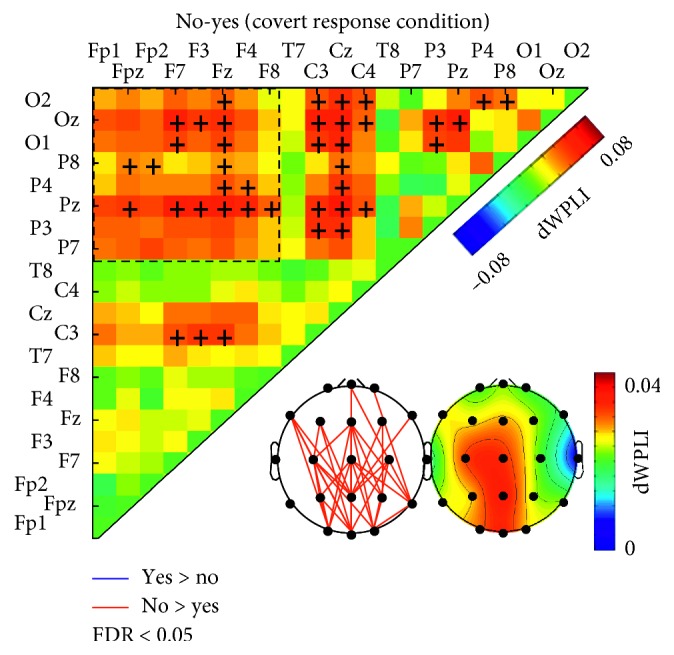
Adjacency matrix shows the differences of the dWPLI between “yes” and “no” (averaged within the significant TF cluster obtained in Figure 4(a)) for covert response. The black crosses denote significant differences between “yes” and “no,” determined by the mass-univariate method (FDR<0.05). Black dotted boxes indicate the electrode pairs between frontal and posterior (parietal and occipital) regions. Lower panels show the patterns of connections between electrode pairs corresponding to significant difference between “yes” and “no” (red: “no”>yes,” blue: “no”<“yes”) and topographical distributions of the “yes/no” difference in dWPLI.

Statistically significant differences were identified only for the covert response condition in 40 electrode pairs (indicated by “+” in Figure 5), which were found mostly in anterior-posterior connections, among electrodes located over parietal and other regions. The alpha-band dWPLI was higher for “no” than for “yes” for all the electrode pairs with significant differences.

## 4. Discussion

In this study, we identified the brain activities showing significant differences between the intentions on “yes” and “no,” generated in response to self-referential questions. In particular, the local and global neural synchronies in the alpha-band were significantly different between “yes” and “no,” which were higher for “no” than for “yes.” These substantial differences were identified after the semantics-related processing in response to self-referential questions, only when the covert response was induced. These findings indicate that alpha-band neural synchronies at a critical temporal period may be significantly influenced by intentions in response to self-referential questions.

A significant “yes/no” difference in the alpha-band power was observed at the ∼600–1000 ms period only in the covert response condition. The covert response condition required the subjects to retain the intentions on the response in their mind until the response cue onset. Thus, we speculated that these alpha-band neural synchronies indicate the mental representation during the intention retention, and they are significantly differentiated according to the contents of the intentions.

In our recent ERP study, we identified that the integration of semantic and autobiographical information processing precedes answering “yes” or “no” in response to self-referential questions [6]. We interpreted that the posterior N400 (at ∼300–500 ms) ERP may reflect this integrated information processing which enables the detection of autobiographical fact violation in the question and decision of the answer. This finding on ERP suggests that the neural activities directly reflecting intention, i.e., showing differences between intentions to answer “yes” and “no,” would occur after the temporal period of N400. Thus, we expected that the intention-related difference would be found at a rather late period. The results on alpha-band neural synchrony are in line with this assumption, in that the difference was found in the 600–1000 ms period.

The significant “yes/no” difference in the alpha-band power was identified in the upper alpha frequency range (i.e., 11–13 Hz). Several previous studies have suggested the different functional roles between the lower and upper alpha rhythms during cognitive processes [7, 28, 29]. For example, the lower alpha event-related desynchronization (ERD) (about 7–10 Hz) was related to attentional demands such as alertness and expectancy, whereas the upper alpha ERD (about 10–13.5 Hz) was associated with sensory semantic processing [7, 28, 29]. Furthermore, several studies on working memory have consistently reported that an increased power in the upper alpha frequency range was found while the previously encoded items were retained [7, 10]. This upper alpha ERS related to WM retention was thought to reflect active inhibition of nontask related neural activities and competing sensory or cognitive processes [7, 10].

The alpha-band activity was significantly greater for “no” compared to “yes”. This finding can be interpreted in line with several studies that showed the increases in the alpha-band neural synchrony as a common pattern of neural activity for WM [7, 9–11]. They have provided convergent evidence that the linear increase of alpha-band power occurs with the increasing memory load during WM retention. The increased alpha rhythm can be interpreted as reflecting active inhibition to prevent the inflow of information to cortical regions relevant to maintaining the WM contents [7, 9–11]. Hence, the greater increase in alpha-band activity for “no” in the covert response condition may reflect the increased WM load during the intention retention. In the Korean language, “yes” is one-character word, “네,” and “no” is three-character word, “아니오.” It is plausible that the higher WM load is required to represent intention to respond “no” than “yes” due to the length of the Korean words, which resulted in the higher alpha rhythm. This assumption is supported by an ERP study which reported that greater alpha-band power was induced for retaining longer word [30].

Another interpretation would be to assume that increased alpha-band activity is related to higher attentional demand [31, 32]. In the covert response condition, we identified greater alpha-band activity for “no” compared to “yes,” particularly in the electrodes located over right centroparietal region. Benedek et al. [33] recently reported that a higher alpha rhythm was identified in the right parietal cortex for a higher internal attention condition during a divergent thinking task. They interpreted that the right centroparietal alpha rhythm reflects the deactivation of the right temporoparietal region. The right temporoparital region was suggested to be involved in bottom-up attention, preventing reorienting to the irrelevant input during the task [34]. The deactivation of the right temporoparietal region for the higher attentional condition was shown by several fMRI studies [35, 36]. According to this interpretation, our result of greater alpha rhythm in the electrodes over right parietal area for “no” than for “yes” may imply that a stronger inhibition of outer stimuli by the bottom-up attention network is required for “no” and that intention retention for answering “no” induces a higher internal attentional demand compared to answering “yes”. Several psychophysical studies have suggested that saying “no” requires more effortful reconsideration after comprehending a sentence, and this was supported by psychophysical studies which reported that a longer response time was required for saying “no” than saying “yes” [16, 17]. Accordingly, it is reasonable that a higher internal attentional demand was required for answering “no” than “yes,” due to the stronger reconsideration after the comprehension of the question.

We identified a significant difference in interregional PS in the alpha-band between “yes” and “no” and that the difference was observed prominently among the electrodes located over anterior and posterior regions. This finding may indicate that the mental representation of the intention retention is reflected in the anterior-posterior alpha-band neural synchrony as well as the local alpha-band activity. Two interpretations of greater alpha rhythm for “no” that we suggested above imply that stronger inhibition is required for “no” due to higher cognitive load. Several previous studies have postulated that this top-down inhibitory control is enabled by the frontal area [7, 11, 37]. Moreover, top-down processing has been proposed to be mediated by network activities of multiple distant regions synchronized in the alpha- and theta-bands [14]. Thus, it is reasonable to expect distinct differences between “yes” and “no” in interregional PS between frontal and other areas.

The difference in interregional PS between “yes” and “no” was found most apparently among the connections with the electrodes located over parietal area (Figure 5). This is comparable to the local alpha power showing the greatest difference between “yes” and “no” in the electrodes over the parietal region. Taken together, the local and global neural synchronies in the alpha-band suggest that different cognitive loads are required for “yes” and “no” intentions. Previously, it was suggested that the frontoparietal interaction in the alpha-band is involved in the regulation of top-down modulation for object representations in WM by neural ensemble, which becomes strengthened with memory load increase [11, 15]. The parietal region was considered as a major hub in the alpha-band frontoparietal network, linking attentional and sensory processing [15, 37]. In line with these findings, we interpreted that the stronger anterior-posterior PS in the alpha-band for “no” may reflect that a stronger top-down inhibition of sensory processing, probably enabled by frontal area, is required to retain the intention to answer “no” than “yes.”

There are a few limitations in this study. The covert response condition does not include any behavioral response recording. This means that we cannot perfectly rule out the possibility of errors in the answers to the questions. However, we consider that all the subjects did not make any mistakes or errors. The questions were generated from a pretest using the questionnaire based on self-referential information, which means that the subjects were familiar with the contents of the questions. Furthermore, immediately after the experiment, we verified that there was neither vague question to answer nor any incorrect answer. Another limitation is that the volume conduction problem prohibits a rigorous functional connectivity (FC) analysis to investigate interaction among cortical regions using scalp EEGs [38]. Also the FC analysis using scalp EEG may yield spurious results emphasizing connections between neighboring regions [39]. To overcome this limitation, we adopted the dWPLI which is known to be robust to the volume conduction effect [23]. We confirmed that the volume conduction effect could be alleviated in that the correlation between the connectivity strength and interelectrode distance was not observable (the results are not provided here). Nevertheless, it remains impossible to infer the location of cortical sources which contributed to the FC. Further studies on the FC among cortical sources may be required based on EEG source localization [40].

Overall, our experimental task consists of two temporally distinct steps. First, the decision of “yes” or “no” is made, which may be generated automatically by the question and reflected in N400 ERP component (at ∼300–500 ms) [6]. Next, the information on the decision should be retained in WM until the “Respond” cue appears, which is associated with the oscillatory neural activities in alpha-band (at ∼600–1000 ms). Our findings suggest that both local and global neural synchronies in the alpha-band are significantly differentiated during a critical temporal epoch, according to the contents of the mental representation of the intention.

One of the most important technological bases of BCI is to read or “decode” the users' intention from their brain activities. Two approaches have been pursued for the intention decoding for BCI: (1) using voluntary self-regulation of specific brain signals such as slow cortical potential [41] and sensorimotor rhythms [42]; (2) using evoked brain activities such as P300 ERP [43, 44] and steady-state evoked potential [45, 46]. Both were not so successful for the CLIS patients, presumably due to the extinction of goal-directed cognition and thought in CLIS patients [47]. An alternative approach is necessary, which does not involve volitional and highly cognitive efforts. For example, Birbaumer and colleagues suggested an approach based on classical conditioning [5, 48, 49]. They tried to associate language stimuli with unpleasant sensory stimuli so that cortical responses to these nonlanguage stimuli are conditioned according to the language stimuli [5, 48, 49]. This is remarkable considering that language is the most natural means of communication. However, the most fundamental linguistic communication consists of questions and answers. The simplest one is binary “yes or no” question and answer, which enables fundamental interpersonal communication (e.g., “Is your name John?,” “Yes.” or “Do you want to drink water?,” “No.”). By decoding the intentions to answer either “yes” or “no” from brain activities, a more natural BCI, which does not require any operant training or heavy cognitive efforts, may be implemented. Our results in this paper provides a neurophysiological basis toward this goal, since the decoding of “yes” and “no” from single-trial EEGs may be plausible. It is not possible to utilize the experimental paradigm in this study to the BCI without any changes, and thus, further study is necessary.

## Data Availability

The data used to support the findings of this study have not been made available because some participants of this study did not agree to distribute their physiological signals.
